# RNAcentral: a hub of information for non-coding RNA sequences

**DOI:** 10.1093/nar/gky1034

**Published:** 2018-11-05

**Authors:** The RNAcentral Consortium, Blake A Sweeney, Anton I Petrov, Boris Burkov, Robert D Finn, Alex Bateman, Maciej Szymanski, Wojciech M Karlowski, Jan Gorodkin, Stefan E Seemann, Jamie J Cannone, Robin R Gutell, Petra Fey, Siddhartha Basu, Simon Kay, Guy Cochrane, Kostantinos Billis, David Emmert, Steven J Marygold, Rachael P Huntley, Ruth C Lovering, Adam Frankish, Patricia P Chan, Todd M Lowe, Elspeth Bruford, Ruth Seal, Jo Vandesompele, Pieter-Jan Volders, Maria Paraskevopoulou, Lina Ma, Zhang Zhang, Sam Griffiths-Jones, Janusz M Bujnicki, Pietro Boccaletto, Judith A Blake, Carol J Bult, Runsheng Chen, Yi Zhao, Valerie Wood, Kim Rutherford, Elena Rivas, James Cole, Stanley J F Laulederkind, Mary Shimoyama, Marc E Gillespie, Marija Orlic-Milacic, Ioanna Kalvari, Eric Nawrocki, Stacia R Engel, J Michael Cherry, SILVA Team, Tanya Z Berardini, Artemis Hatzigeorgiou, Dimitra Karagkouni, Kevin Howe, Paul Davis, Marcel Dinger, Shunmin He, Maki Yoshihama, Naoya Kenmochi, Peter F Stadler, Kelly P Williams

**Affiliations:** 1 European Molecular Biology Laboratory, European Bioinformatics Institute, Wellcome Genome Campus, Hinxton, Cambridge CB10 1SD, UK; 2 Department of Computational Biology, Institute of Molecular Biology and Biotechnology, Adam Mickiewicz University, Poznan, Poland; 3 Center for non-coding RNA in Technology and Health, Department of Veterinary and Animal Sciences, University of Copenhagen, Frederiksberg, Denmark; 4 Institute for Cellular and Molecular Biology, and the Center for Computational Biology and Bioinformatics, The University of Texas at Austin, Austin, TX 78712, USA; 5 dictyBase, Northwestern University, 420 E. Superior St., Chicago, IL 60611, USA; 6 Department of Molecular and Cellular Biology, Harvard University, Biological Laboratories, 16 Divinity Avenue, Cambridge, MA 02140, USA; 7 Department of Physiology, Development and Neuroscience, University of Cambridge, Downing Street, Cambridge CB2 3DY, UK; 8 Institute of Cardiovascular Science, University College London, London, UK; 9 Department of Biomolecular Engineering, University of California, Santa Cruz, CA, USA; 10 DIANA-Lab, Department of Electrical & Computer Engineering, University of Thessaly, 382 21 Volos, Greece; 11 Hellenic Pasteur Institute, 127 Vasilissis Sofias Avenue, 11521 Athens, Greece; 12 Ghent University and Cancer Research Institute Ghent, 9000 Ghent, Belgium; 13 St Vincent's Clinical School, UNSW Sydney, Sydney, Australia; 14 BIG Data Center, Beijing Institute of Genomics, Chinese Academy of Sciences, Beijing 100101, China; 15 Faculty of Biology, Medicine and Health, The University of Manchester, Manchester, UK; 16 International Institute of Molecular and Cell Biology in Warsaw, Warsaw, Poland; 17 Jackson Laboratory, 600 Main St., Bar Harbor, ME 04609, USA; 18 Key Laboratory of RNA Biology, Institute of Biophysics, Chinese Academy of Sciences, Beijing 100101, China; 19 Institute of Computing Technology, Chinese Academy of Sciences, Beijing 100080, China; 20 Cambridge Systems Biology and Department of Biochemistry, University of Cambridge, Sanger Building, 80 Tennis Court Road, Cambridge, Cambridgeshire CB2 1GA, UK; 21 Department of Plant, Soil and Microbial Sciences, Michigan State University, East Lansing, MI 48824, USA; 22 College of Pharmacy and Health Sciences, St John's University, Queens, NY 11439, USA; 23 Ontario Institute for Cancer Research, Toronto, ON M5G 0A3, Canada; 24 National Center for Biotechnology Information, U.S. National Library of Medicine, Bethesda, MD 20894, USA; 25 Department of Biomedical Engineering, Medical College of Wisconsin and Marquette University, Milwaukee, WI 53226, USA; 26 Department of Genetics, Stanford University, Palo Alto, CA 94304 USA; 27 Microbial Genomics and Bioinformatics Research Group, Max Planck Institute for Marine Microbiology, D-28359 Bremen; 28 Jacobs University Bremen, School of Engineering and Science, D-28759 Bremen; 29 Frontier Science Research Center, University of Miyazaki, Miyazaki, Japan; 30 Phoenix Bioinformatics, Fremont, CA 94538, USA; 31 Systems Biology Department, Sandia National Laboratories, Livermore, CA 94551, USA; 32 Bioinformatics Group, Department of Computer Science, and Interdisciplinary Centre for Bioinformatics, Leipzig University, Härtelstr. 1618, 04107 Leipzig, Germany; 33 Competence Center for Scalable Data Services and Solutions Dresden/Leipzig, German Centre for Integrative Biodiversity Research (iDiv), and Leipzig Research Center for Civilization Diseases, Universität Leipzig, Ritterstrasse 9–13, 04109 Leipzig, Germany; 34 Max Planck Institute for Mathematics in the Sciences, Insel Strasse 22, 04103 Leipzig, Germany; 35 Fraunhofer Institute for Cell Therapy and Immunology, Perlickstrasse 1, 04103 Leipzig, Germany; 36 Department of Theoretical Chemistry, University of Vienna, Wahringerstrasse 17, 1090 Vienna, Austria; 37 Center for RNA in Technology and Health, University of Copenhagen, Grønnegårdsvej 3, Frederiksberg C, Denmark; 38 Santa Fe Institute, 1399 Hyde Park Road, Santa Fe, NM 87501, USA

## Abstract

RNAcentral is a comprehensive database of non-coding RNA (ncRNA) sequences, collating information on ncRNA sequences of all types from a broad range of organisms. We have recently added a new genome mapping pipeline that identifies genomic locations for ncRNA sequences in 296 species. We have also added several new types of functional annotations, such as tRNA secondary structures, Gene Ontology annotations, and miRNA-target interactions. A new quality control mechanism based on Rfam family assignments identifies potential contamination, incomplete sequences, and more. The RNAcentral database has become a vital component of many workflows in the RNA community, serving as both the primary source of sequence data for academic and commercial groups, as well as a source of stable accessions for the annotation of genomic and functional features. These examples are facilitated by an improved RNAcentral web interface, which features an updated genome browser, a new sequence feature viewer, and improved text search functionality. RNAcentral is freely available at https://rnacentral.org.

## INTRODUCTION

**Figure 1.**
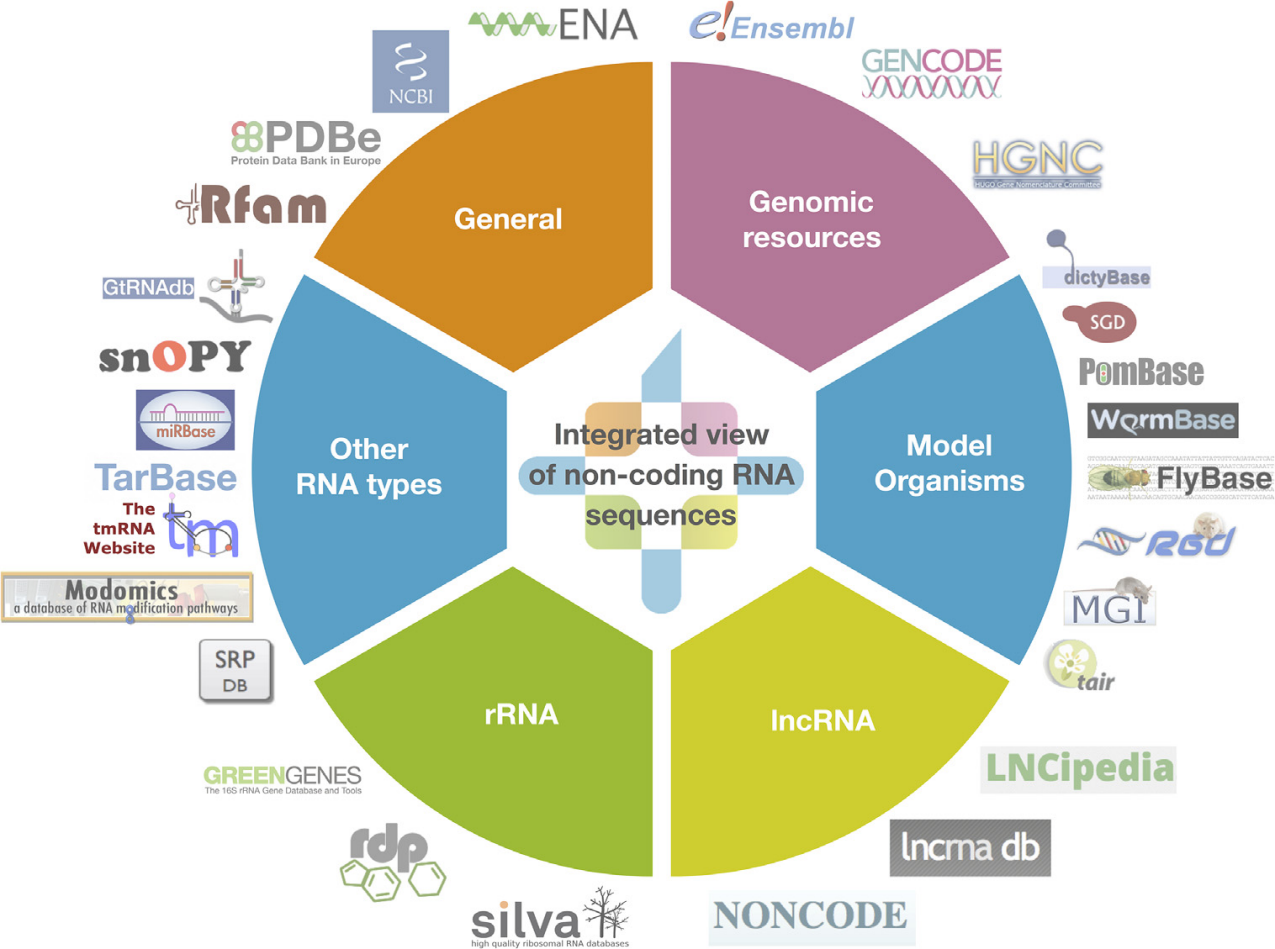
A diagram showing the 28 Expert Databases imported into RNAcentral as of September 2018, organised according to their contents. The full list of databases is available at https://rnacentral.org/expert-databases.

RNAcentral is a comprehensive database of ncRNA sequences from a broad range of species (1). Launched in 2014 (2), RNAcentral provides unified access to the data from 28 different RNA resources, known as Expert Databases (Figure 1).

The primary objective of the RNAcentral database is to provide a comprehensive set of high quality ncRNA sequences to the widest possible audience. **U**nique **R**NA **s**equences are assigned ‘URS’ identifiers, which become the primary entities around which all information is stored, integrated from multiple different sources, and presented. Relevant data (e.g. accessions, genomic locations, functional annotations) for each ncRNA are then displayed within individual sequence pages on the website. The RNAcentral website has four main functionalities:

1. **Text search**: allows users to search and compare ncRNA sequences from different databases.
2. **Sequence search** (powered by nhmmer (3)): users may search any nucleotide sequence for similarity to known ncRNA sequences from contributing databases.
3. **Genome browser**: allows users to view ncRNA annotations in a genomic region of interest.
4. **Bulk data download**: all data are accessible via the FTP archive and for programmatic data access via an API (https://rnacentral.org/downloads).

In this paper we discuss the recent improvements and changes that have expanded RNAcentral's abilities to serve scientists with various backgrounds and data needs. Since the last RNAcentral publication (1), the database has provided 5 releases (versions 6–10) and now imports ncRNA data from 28 databases (seven additional databases since 2017). In addition to increasing the number of sequences, we have added several new data types, including: (1) genomic locations for sequences in selected model organisms, (2) quality control information for all sequences, (3) functional and structural annotations, and (3) miRNA targets. These new data and the improvement in the website functionality are described in detail below.

## COMPREHENSIVE GENOME MAPPING

The genomic context of a particular ncRNA can provide important clues pertaining to its function. For example, the location of both long and short RNAs in the Hox cluster of bilaterian animals implicates those RNAs in key developmental processes (4,5). Genomic context can also reveal potential antisense RNAs to their targets. Previously, RNAcentral provided genomic locations for sequences only if the expert databases submitted the coordinates. However, many databases do not capture genomic coordinates on the latest genome assemblies, or indeed at all. Due to this limitation in release 9 23.6% of human ncRNA sequences had no annotated genomic location.

To overcome this limitation, we have developed a comprehensive approach to map RNAcentral sequences to their genome locations. We downloaded 296 genomes from Ensembl (6) and all Ensembl Genomes (7) divisions except Bacteria (due to scale). For each species, all RNAcentral sequences that did not already have a genome mapping were mapped to the corresponding genome using blat (8). Exact matches, defined as alignments with an edit distance of zero, were stored. For sequences that did not have an exact match (∼14% of all ncRNAs across all genomes), hits with at least 95% sequence identity were retained. To minimise the chance of spurious hits, we limit the length of insertions for sequences shorter than 100 nucleotides. We evaluated this pipeline by mapping all ncRNA sequences from Ensembl and found it successfully recovered the correct location for >99% indicating it is accurate.

After applying this mapping pipeline, the number of sequences with reported genome mappings has increased by a factor of 10 across all species, now providing sequence locations for > 95% from the sequences of many important model organisms (Table 1). The mapping will be updated with each RNAcentral release using the latest genome versions from Ensembl, ensuring that these mappings are always up-to-date.

**Table 1.** The increase in the number of sequences with genome coordinates across key species

Species	Genome assembly	Sequence count	Percent mapped	Improvement	Percent of sequences with more than one mapping
*Caenorhabditis elegans*	WBcel235	27 137	99.7%	4.9%	2.4%
*Dictyostelium discoideum*	dicty_2.7	167	99.4%	9.6%	25.3%
*Homo sapiens*	GRCh38.p12	204 847	99.1%	23.6%	7.2%
*Rattus norvegicus*	Rnor_6.0	131 013	97.7%	70.6%	15.0%
*Mus musculus*	GRCm38.p6	182 379	96.4%	47.1%	9.4%
*Schizosaccharomyces pombe*	ASM294v2	2196	96.4%	15.8%	12.2%
*Drosophila melanogaster*	BDGP6	6630	96.1%	34.4%	22.0%

Users can explore genomic mapping in the context of Ensembl genes and transcripts either on sequence report pages or by navigating to any genome location using the RNAcentral genome browser (https://rnacentral.org/genome-browser). Additionally, GFF3 and BED files can be downloaded from the RNAcentral FTP archive.

**Figure 2.**
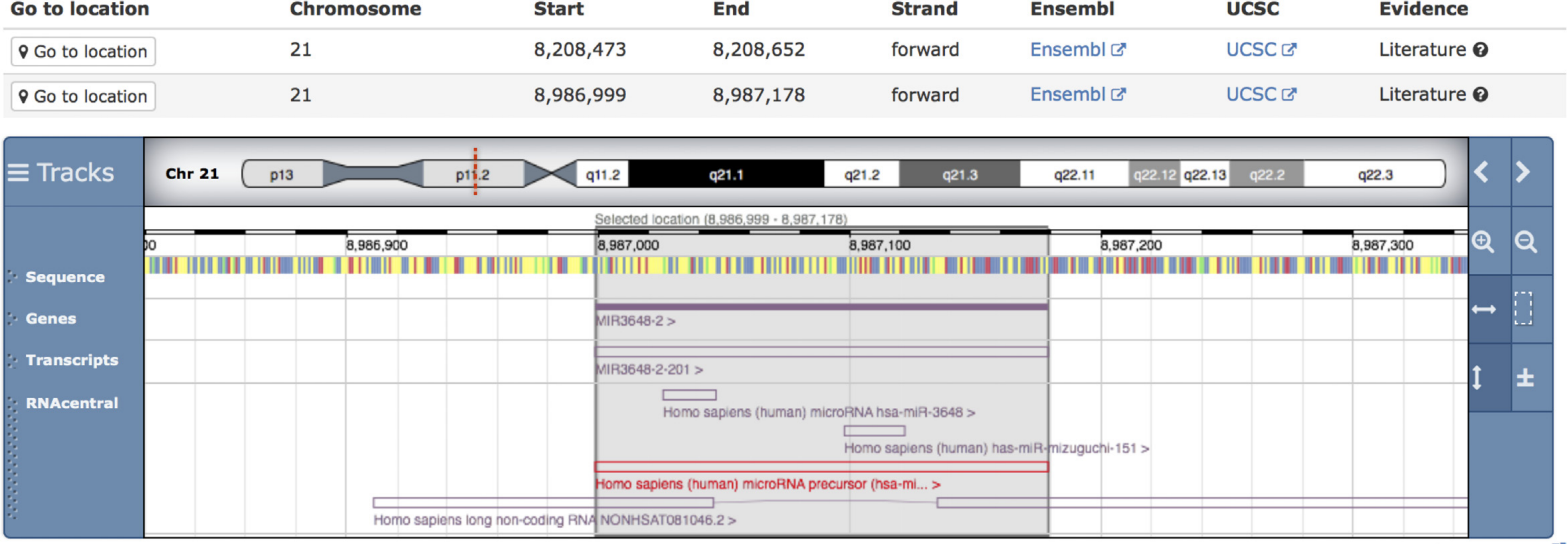
Novel snoRNA Me18S-G1506 (URS0000A59F5E_7227) found in the ENA database that was mapped with 100% sequence identity to the *Drosophila melanogaster* genome using the new genome mapping pipeline.

Genome mapping of ncRNAs within RNAcentral can identify inconsistencies between data sources and thereby facilitate improvements in ncRNA annotations across expert databases. For example, 10 out of 11 novel *D. melanogaster* snoRNAs described in a recent paper (9) and submitted to the INSDC (10) do not overlap with existing annotated ncRNAs (Figure 2), making these features candidates for review by databases such as FlyBase (11), snoPY (12) and Rfam (13). RNAcentral is developing a pipeline for systematic identification of such annotation anomalies and alerting the relevant databases.

## QUALITY CONTROLS USING RFAM

RNAcentral aims to provide a comprehensive and high-quality set of ncRNA sequences. In order to accomplish this, we have developed a pipeline to implement quality checks based on Rfam classification of RNA families (13). All RNAcentral sequences were searched against all Rfam families using the Infernal software (14). Although Rfam does not include piRNAs, full-length lncRNAs, and several other ncRNA types (13), the majority of RNAcentral sequences (80%) are matched by one or more Rfam families, demonstrating that classification by Rfam provides broad quality control coverage. The remaining 20% of sequences in RNAcentral that do not match an Rfam family are primarily (60%) from RNA types that Rfam does not model (piRNA, mature miRNAs, lncRNAs) or from a generic biotypes such as other or miscellaneous RNA.

This analysis produces a series of warnings which are displayed in search results and on sequence pages. Currently, RNAcentral provides three types of warnings. (i) Potential contamination: triggered when a eukaryotic sequence matches an Rfam family that is only found in bacteria, which could indicate either bacterial contamination or taxonomic misclassification. (ii) Incomplete sequences: triggered when an RNAcentral sequence matches only a small part (<50%) of an Rfam model. (iii) Potential misannotations: triggered when either an rRNA or tRNA sequence does not match the corresponding Rfam families.

The distribution of warnings by type is shown in Table 2. The majority (60%) of sequences do not have any warnings. Of those with warnings, most (34% of all sequences) are incomplete sequences. The majority of incomplete sequences are partial rRNAs (5 070 967 or 99%) followed by tRNAs (<1%) and other RNA types (<1%).

**Table 2.** The number of sequences with and without Rfam warnings

Warning type	Number of sequences
No problems detected	9 055 240 (60%)
Incomplete sequence	5 074 317 (34%)
Potential misannotation	778 974 (5%)
Potential contamination	162 562 (1%)

The warnings provided by this quality control are searchable on the browse page, using the ‘QC warning found’ filter on the lower left. For details on searching please refer to the RNAcentral search help at: https://rnacentral.org/help/text-search. Additionally, RNAcentral provides a flat file of all Rfam annotations in the FTP archive (ftp://ftp.ebi.ac.uk/pub/databases/RNAcentral/current_release/rfam/). The file can be used by expert databases to add Rfam links or validate existing RNA annotations by checking if they match the expected Rfam families. It is important to interpret the results of this automatic quality control analysis with caution. For example, eukaryotic sequences found in organelles are expected to match bacterial Rfam models, so the warnings are only a guide to potential issues.

## NEW DATA AND FUNCTIONAL ANNOTATIONS

In this section we highlight the new data RNAcentral has imported since last publication. This data not only includes more ncRNA sequences, but also new types of information such as tRNA secondary structures and high-quality miRNA/mRNA interactions.

### New expert databases

Since our last publication, RNAcentral has imported ncRNA data from seven new databases, including three Model Organism Databases (MODs): **FlyBase** (11), Mouse Genome Informatics (**MGI**) (15), and the Rat Genome Database (**RGD**) (16). The MODs contribute high-quality, manually reviewed ncRNAs for the species they represent, thereby adding significant value to RNAcentral.

Additionally, we have imported ncRNA data from **Ensembl** (6), **GENCODE** (17), **HGNC** (HUGO Gene Nomenclature Committee) (18), and **TarBase** (19). Ensembl provides automated RNA gene annotations for over 62 vertebrate genomes predicted based on Ensembl ncRNA and lincRNA pipeline (20), while GENCODE provides high quality manual annotations for large lncRNAs found in human and mouse genomes.

### tRNA secondary structures imported from GtRNAdb

**Figure 3.**
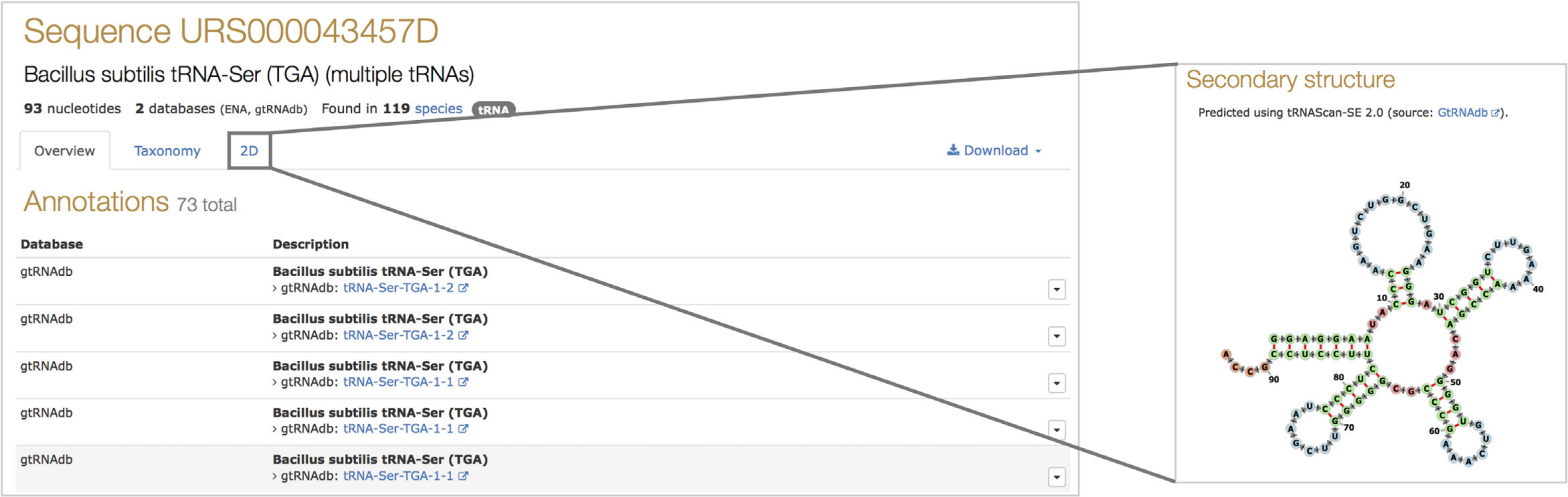
Example secondary structure of tRNA-Ser-TGA-1-2 from *Bacillus subtilis* (URS000043457D_1423) visualized using Forna. The nucleotides in the Forna diagram are colored by secondary structure element, with helixes in green, hairpin loops in blue and red otherwise.

Following a major upgrade of the tRNAscan-SE software, Genomic tRNA Database (GtRNAdb) (21) now provides a much broader range of tRNA sequences, including tRNAs with possible introns. RNAcentral has imported bacterial, archaeal, fungal, as well as human, rat and mouse sequences from GtRNAdb increasing the coverage from 382 species to 4239. RNAcentral also displays RNA secondary structures provided by GtRNAdb using Forna (22) (Figure 3). This is the first secondary structure dataset integrated into RNAcentral.

### miRNA target interactions from TarBase

**Figure 4.**
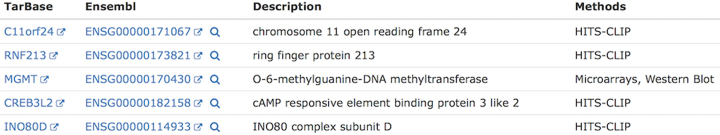
New section of the sequence report pages showing target proteins for miRNA hsa-miR-612 (URS0000759916_9606). The table provides links to Ensembl genes and TarBase summary pages and shows experimental methods.

RNAcentral also imported its first intermolecular interactions data from TarBase v8 (19). TarBase provides hundreds of thousands of experimentally supported microRNA (miRNA) targets derived from >30 experimental methodologies applied to ∼600 cell type/tissues. The integrated dataset incorporates 1507 distinct miRNAs from human and mouse, annotating 559 000 miRNA:gene pairs, corresponding to 33 858 protein coding targets. The interactions are displayed on sequence report pages (Figure 4) and can be queried using the text search.

## OTHER IMPROVEMENTS

In addition to new data, RNAcentral has also improved several aspects of the website based upon extensive user feedback. Here, we discuss improvements to the search interface, sequence descriptions, a new sequence feature viewer, as well as a JSON-based submission pipeline.

### More informative search results

**Figure 5.**
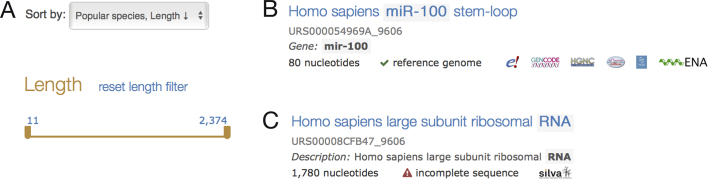
(**A**) New text search interface options for filtering sequences by length (bottom) or changing the order of the results (top). (**B**) Structured snippets in text search results. The string matching the query (‘mir-100’) is highlighted in light-gray. The logos of expert databases annotating the sequence are displayed and additional information about the databases can be viewed on mouse hover. (**C**) A search result showing a sequence with a quality check failure. Here the red warning symbol indicates the sequence has an error, along with the type of issue detected, incomplete sequence here.

The text search interface has been substantially improved based on user feedback. First, the search now features a text **autocomplete** functionality. Secondly, the search supports more filtering options. These include: length, which helps identify only complete sequences; and the new quality checks, which allow users to limit search results to only those sequences without warnings. Finally, it is also now possible to **sort search results by length** in ascending or descending order (Figure 5A).

Search results now provide structured snippets (Figure 5B, C). These snippets are a concise summary of the matched sequence showing the gene symbols, sequence length, and a list of databases providing annotation for the entry, as well as any quality check issues. The snippet also explains why the entry is shown by highlighting the matched text (Figure 5B).

### Improved sequence descriptions

RNAcentral provides descriptions for all sequences, which are displayed in search results as a summary and on sequences pages. Informative descriptions help to quickly identify sequences of interest among other search results (Figure 5B). RNAcentral has created a rule-based system to take into account expert database annotations to select an informative description for each sequence.

In some cases, RNAcentral generates new descriptions to better represent the data from specific databases. For example, sequence URS000075A3E2_9606 is a miRNA encoded at four genome locations, which corresponds to four different descriptions from miRBase. Picking a single description for the unique sequence would not accurately summarize the different locations, so the following description is generated: ‘Homo sapiens (human) microRNA hsa-mir-6859 precursor (hsa-mir-6859 1 to 4)’. As this description shows the full range of precursors that are part of this sequence and is more informative than any one description. The generation of descriptions is done automatically on an ongoing basis.

### Displaying sequence features

**Figure 6.**
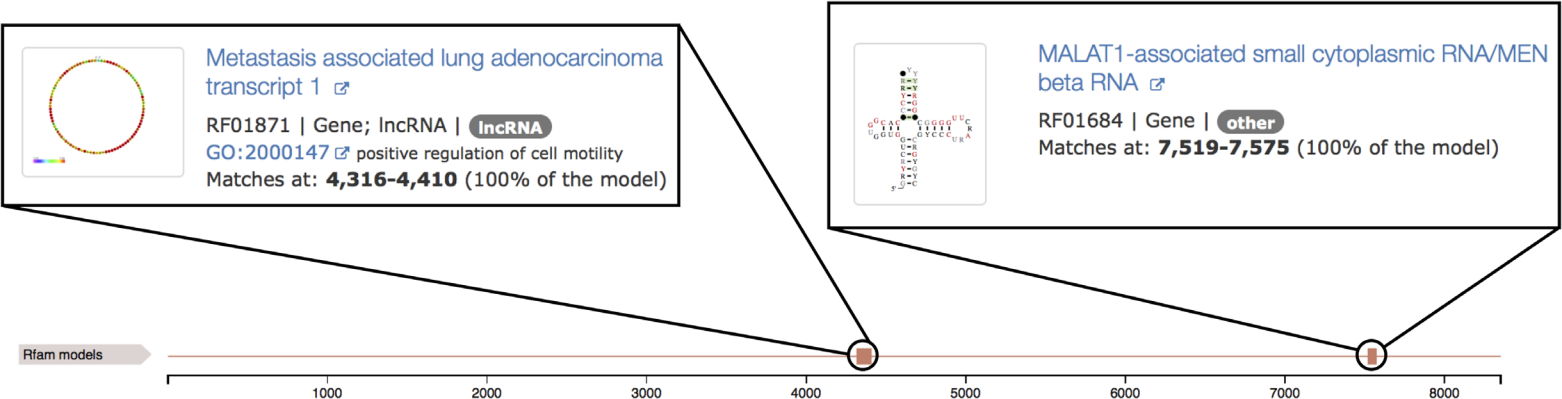
New section of the sequence report showing Rfam annotations. In this example, an ENA sequence URS00005B7DD8_9606, originally annotated as miscellaneous RNA (misc_RNA), matches a conserved domain of the MALAT1 Rfam family (RF01871) and MEN beta RNA (RF01684). The locations of the Rfam matches are shown in the feature viewer.

RNAcentral now contains a sequence feature viewer. This viewer is used to display modifications and Rfam annotations (Figure 6), replacing our previous sequence display with a more informative and accessible summary of annotations on the sequence.

### Automatic assignment of GO terms

RNAcentral sequences are automatically annotated with GO terms, propagated from the matching Rfam covariance models. When a ncRNA sequence is matched to one or more Rfam families, the GO terms associated with the Rfam family are transitively assigned to the ncRNA sequence. More than 10 millions of these annotations are available through QuickGO (22) (https://www.ebi.ac.uk/QuickGO/annotations?assignedBy=RNAcentral), as well as in the Gene Ontology Annotation (GOA) Database (https://www.ebi.ac.uk/GOA). RNAcentral is the largest source of GO annotations for ncRNA sequences. Additionally, RNAcentral identifiers (URS) are used as the basis for GO annotations in GOA and QuickGO. These identifiers were chosen because they provide a stable, precise, and comprehensive method for referring to ncRNA sequences (24).

### New JSON-based submission process

In order to streamline the submission of data to RNAcentral, we defined a new exchange format and validation software. The new system results in a clear and unambiguous protocol for the preparation, validation and submission of ncRNA data and metadata by the expert databases to RNAcentral, and has made the submission process faster, more reliable and flexible.

The current version is based on a corresponding effort by the Alliance of Genome Resources (https://github.com/alliance-genome/agr_schemas), and has been developed with extensive feedback from FlyBase (11), miRBase (25), LNCipedia (26), GtRNAdb (21) and TarBase (19). The schema and a JSON schema validator are available at https://github.com/RNAcentral/rnacentral-data-schema.

## USE OF RNAcentral DATA

Here we describe how being part of the RNAcentral Consortium has helped two expert databases to improve their resources. We also present examples of RNAcentral usage by the research community.

## HGNC canonical human ncRNA gene set

The HGNC (18) is the only international resource that has the authority to approve gene symbols and names for human genes. HGNC began approving symbols for human small non-coding RNA genes in the 1980s, starting with mitochondrial tRNA genes. Since the identification of many new classes of RNA the naming of ncRNA genes has become one of HGNC’s core activities. HGNC collaborates with several RNAcentral expert databases to name specific classes of small ncRNAs, such as miRBase for miRNAs and GtRNAdb for tRNAs. HGNC also names long non-coding RNA (lncRNA) genes by working directly with research groups and genome annotators. The lncRNA gene names are based on reported function wherever possible, and on genomic location where the function is unknown.

Due to its relative completeness, the HGNC ncRNA set was chosen to be the **canonical human gene set** in RNAcentral, meaning HGNC is promoted above other sources of human data. Each HGNC entry is matched to one RNAcentral sequence through cross references to RefSeq, Ensembl, GtRNAdb and other databases that are manually curated by the HGNC. For example, the HGNC entry for HOTAIR corresponds to RefSeq accession NR_003716, which is found in RNAcentral under the identifier URS000075C808.

RNAcentral has helped HGNC by performing **quality control** checks on its data. This enable HGNC to check the mappings between their gene symbols and Ensembl gene annotations and lncRNAdb, and also augment their links to RefSeq transcript sequences. Following a list sent to HGNC from RNAcentral and a resulting discussion with RefSeq gene annotators, HGNC withdrew the gene symbol HPVC1 (gene name: human papillomavirus (type 18) E5 central sequence-like 1) because there was a lack of evidence for transcription at this locus. RefSeq also withdrew their gene entry for HPVC1.

### Functional annotation of miRNAs

Functional annotation of gene products using the Gene Ontology has proven vital for interpretation of scientific studies, especially for large-scale studies where functions and roles of many gene products need to be analysed (27). However, this type of high-quality functional annotation has been lacking for many classes of ncRNAs. There is an abundance of published information about the targets and the functional roles of individual miRNAs in the literature, but that information is not curated or systematically available in any database. Researchers therefore commonly infer functional roles of miRNAs by mining lists of predicted targets (28–30). However, this has been shown to lead to biased and unreliable interpretations of miRNA function (29,30).

The Functional Gene Annotation Team at University College London (UCL) started curating experimentally verified GO terms for mature miRNAs in 2014. However, any slight change in a miRNA sequence can mean that it targets different mRNAs for silencing, and potentially different biological processes and pathways. Therefore, to ensure GO annotations are associated with the correct mature miRNA sequence, stable species-specific database identifiers were required (24). The provision of RNAcentral identifiers has allowed the UCL curators to identify miRNA sequences reported in specific publications unambiguously. Since it is common practice for authors to display an alignment of the mRNA with the targeting miRNA sequence in reverse orientation (3′ to 5′), RNAcentral implemented a ‘reverse sequence and search again’ option into the sequence similarity search tool to assist finding the correct miRNA identifier. Occasionally, authors will only show a partial miRNA sequence in a publication. In these cases, a text search in RNAcentral for the miRNA name will return all ncRNA matches, allowing the biocurator to manually cross-check with the published sequence to determine the correct sequence for GO term assignment.

As discussed above, ncRNA annotations in QuickGO are based upon RNAcentral identifiers, which makes distributing UCL GO annotations simple. UCL annotations are provided to several high-profile knowledgebases such as Ensembl, NCBI Gene, miRBase, as well as the GO Consortium. Additionally, the experimentally validated interactions between the mature miRNA and its targets are provided as a PSICQUIC web service (http://www.ebi.ac.uk/Tools/webservices/psicquic/view/main.xhtml), named ‘EBI-GOA-miRNA, enabling this data to be used in interaction networks (28).

**Figure 7.**
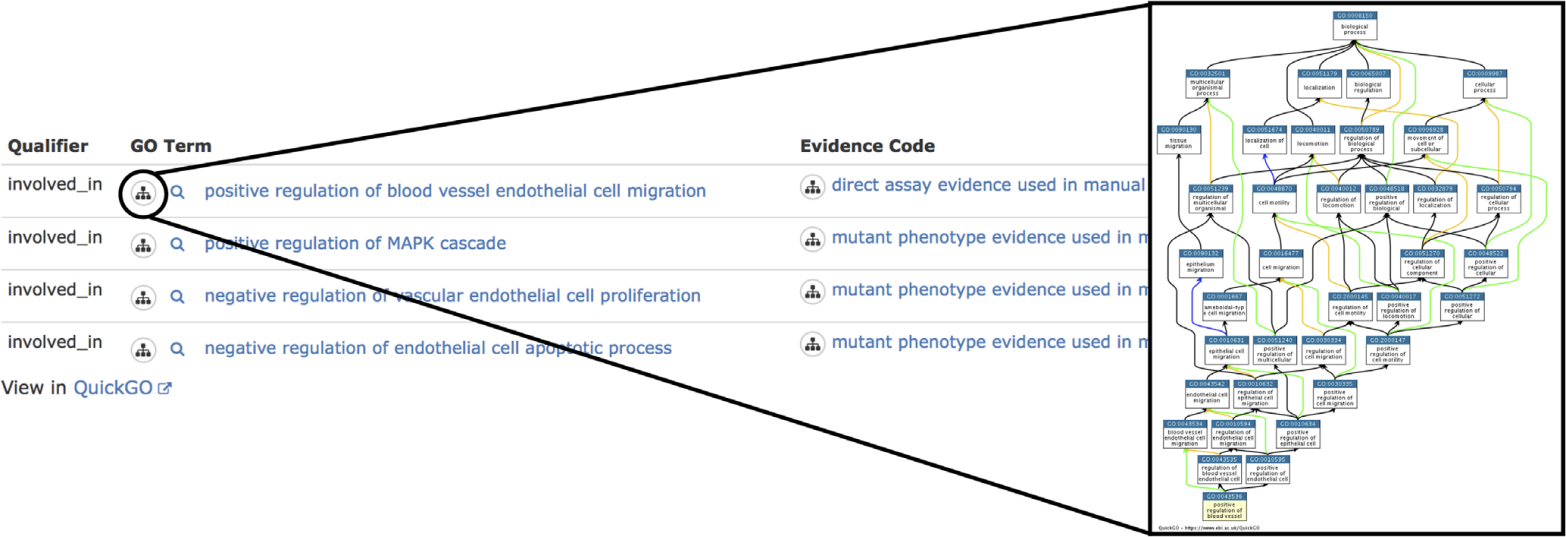
RNAcentral visualization of GO annotations for miRNA hsa-mir-126 (URS0000759B6D_9606) that is involved in heart development.

The UCL team has created over 5,000 GO annotations for over 570 miRNAs from human, mouse and rat. These GO annotations are now displayed in the RNAcentral entries for the mature miRNAs (Figure 7), with links out to the QuickGO browser (23) for the full annotation records. The consistent use of RNAcentral identifiers for functional annotation has also facilitated the import and display of miRNA functional data in the miRBase database (25).

### Use of RNAcentral by the wider research community

We monitor RNAcentral usage by analyzing paper citations and engaging with the users online and at conferences. One of the main uses of RNAcentral is as a source of reference data. In several studies RNAcentral sequences from an organism or RNA type of interest are downloaded and then the novel ncRNAs are compared against the RNAcentral sequences to classify them or to determine if the ncRNAs have been observed before. For example, RNAcentral data were used to study miRNA expression in breast cancer (31), to annotate the sea anemone genome with ncRNAs and study miRNA-mediated modulation of the host transcriptome in cnidarian-dinoflagellate symbiosis (32), and to understand the physiological regulation of reproduction in goats (33). Additionally, Ensembl regularly imports identifiers and descriptions from RNAcentral. Currently, in Ensembl, there are 579,783 RNAcentral related entries for over 112 species. RNAcentral data are also used in the private sector where the sequences have been used to build a reference database for metagenomics analysis using the MG7 pipeline by a company called Era7 Bioinformatics (https://era7bioinformatics.com/en/page.cfm?id=464). More use cases can be found on a dedicated web page (https://rnacentral.org/use-cases).

### Future plans

We are currently working on several improvements such as computing and displaying standardized secondary structures using TRAVeLer (34), a faster release procedure, and more extensive quality controls. We expect RNAcentral to continue growing in utility and reach as more features are added and more databases join the consortium. For example, we plan to extend our genome mapping to include Ensembl Bacteria. We are always open to feedback and our contact information is available at https://rnacentral.org/contact.

## DATA AVAILABILITY

RNAcentral is an open source project with all code available in the GitHub organization: https://github.com/rnacentral/.
