# RNAcentral: a hub of information for non-coding RNA sequences

**DOI:** 10.1093/nar/gky1206

**Published:** 2018-12-08

**Authors:** The RNAcentral Consortium, Blake A Sweeney, Anton I Petrov, Boris Burkov, Robert D Finn, Alex Bateman, Maciej Szymanski, Wojciech M Karlowski, Jan Gorodkin, Stefan E Seemann, Jamie J Cannone, Robin R Gutell, Petra Fey, Siddhartha Basu, Simon Kay, Guy Cochrane, Kostantinos Billis, David Emmert, Steven J Marygold, Rachael P Huntley, Ruth C Lovering, Adam Frankish, Patricia P Chan, Todd M Lowe, Elspeth Bruford, Ruth Seal, Jo Vandesompele, Pieter-Jan Volders, Maria Paraskevopoulou, Lina Ma, Zhang Zhang, Sam Griffiths-Jones, Janusz M Bujnicki, Pietro Boccaletto, Judith A Blake, Carol J Bult, Runsheng Chen, Yi Zhao, Valerie Wood, Kim Rutherford, Elena Rivas, James Cole, Stanley J F Laulederkind, Mary Shimoyama, Marc E Gillespie, Marija Orlic-Milacic, Ioanna Kalvari, Eric Nawrocki, Stacia R Engel, J Michael Cherry, SILVA Team, Tanya Z Berardini, Artemis Hatzigeorgiou, Dimitra Karagkouni, Kevin Howe, Paul Davis, Marcel Dinger, Shunmin He, Maki Yoshihama, Naoya Kenmochi, Peter F Stadler, Kelly P Williams

**Affiliations:** 1 European Molecular Biology Laboratory, European Bioinformatics Institute, Wellcome Genome Campus, Hinxton, Cambridge CB10 1SD, UK; 2 Department of Computational Biology, Institute of Molecular Biology and Biotechnology, Adam Mickiewicz University, Poznan, Poland; 3 Center for non-coding RNA in Technology and Health, Department of Veterinary and Animal Sciences, University of Copenhagen, Frederiksberg, Denmark; 4 Institute for Cellular and Molecular Biology, and the Center for Computational Biology and Bioinformatics, The University of Texas at Austin, Austin, TX 78712, USA; 5 dictyBase, Northwestern University, 420 E. Superior St., Chicago, IL 60611, USA; 6 Department of Molecular and Cellular Biology, Harvard University, Biological Laboratories, 16 Divinity Avenue, Cambridge, MA 02140, USA; 7 Department of Physiology, Development and Neuroscience, University of Cambridge, Downing Street, Cambridge CB2 3DY, UK; 8 Institute of Cardiovascular Science, University College London, London, UK; 9 Department of Biomolecular Engineering, University of California, Santa Cruz, CA, USA; 10 DIANA-Lab, Department of Electrical & Computer Engineering, University of Thessaly, 382 21 Volos, Greece; 11 Hellenic Pasteur Institute, 127 Vasilissis Sofias Avenue, 11521 Athens, Greece; 12 Ghent University and Cancer Research Institute Ghent, 9000 Ghent, Belgium; 13 St Vincent's Clinical School, UNSW Sydney, Sydney, Australia; 14 BIG Data Center, Beijing Institute of Genomics, Chinese Academy of Sciences, Beijing 100101, China; 15 Faculty of Biology, Medicine and Health, The University of Manchester, Manchester, UK; 16 International Institute of Molecular and Cell Biology in Warsaw, Warsaw, Poland; 17 Jackson Laboratory, 600 Main St., Bar Harbor, ME 04609, USA; 18 Key Laboratory of RNA Biology, Institute of Biophysics, Chinese Academy of Sciences, Beijing 100101, China; 19 Institute of Computing Technology, Chinese Academy of Sciences, Beijing 100080, China; 20 Cambridge Systems Biology and Department of Biochemistry, University of Cambridge, Sanger Building, 80 Tennis Court Road, Cambridge, Cambridgeshire CB2 1GA, UK; 21 Department of Plant, Soil and Microbial Sciences, Michigan State University, East Lansing, MI 48824, USA; 22 College of Pharmacy and Health Sciences, St John's University, Queens, NY 11439, USA; 23 Ontario Institute for Cancer Research, Toronto, ON M5G 0A3, Canada; 24 National Center for Biotechnology Information, U.S. National Library of Medicine, Bethesda, MD 20894, USA; 25 Department of Biomedical Engineering, Medical College of Wisconsin and Marquette University, Milwaukee, WI 53226, USA; 26 Department of Genetics, Stanford University, Palo Alto, CA 94304 USA; 27 Microbial Genomics and Bioinformatics Research Group, Max Planck Institute for Marine Microbiology, D-28359 Bremen; 28 Jacobs University Bremen, School of Engineering and Science, D-28759 Bremen; 29 Frontier Science Research Center, University of Miyazaki, Miyazaki, Japan; 30 Phoenix Bioinformatics, Fremont, CA 94538, USA; 31 Systems Biology Department, Sandia National Laboratories, Livermore, CA 94551, USA; 32 Bioinformatics Group, Department of Computer Science, and Interdisciplinary Centre for Bioinformatics, Leipzig University, Härtelstr. 1618, 04107 Leipzig, Germany; 33 Competence Center for Scalable Data Services and Solutions Dresden/Leipzig, German Centre for Integrative Biodiversity Research (iDiv), and Leipzig Research Center for Civilization Diseases, Universität Leipzig, Ritterstrasse 9–13, 04109 Leipzig, Germany; 34 Max Planck Institute for Mathematics in the Sciences, Insel Strasse 22, 04103 Leipzig, Germany; 35 Fraunhofer Institute for Cell Therapy and Immunology, Perlickstrasse 1, 04103 Leipzig, Germany; 36 Department of Theoretical Chemistry, University of Vienna, Wahringerstrasse 17, 1090 Vienna, Austria; 37 Center for RNA in Technology and Health, University of Copenhagen, Grønnegårdsvej 3, Frederiksberg C, Denmark; 38 Santa Fe Institute, 1399 Hyde Park Road, Santa Fe, NM 87501, USA

*Nucleic Acids Res*., 2018, https://doi.org/10.1093/nar/gky1034

This article has been updated to correct an error in the author's name.

